# SHP-2 restricts apoptosis induced by chemotherapeutic agents via Parkin-dependent autophagy in cervical cancer

**DOI:** 10.1186/s12935-018-0505-3

**Published:** 2018-01-15

**Authors:** Dewen Yan, Danyang Zhu, Xiumin Zhao, Jun Su

**Affiliations:** Department of Obstetrics and Gynecology, Taizhou First People’s Hospital, Hengjie Road, No 218, Huangyan, Taizhou, 318020 Zhejiang China

**Keywords:** SHP-2, Apoptosis, Mitochondria damage, Autophagy, Parkin

## Abstract

**Background:**

Autophagy is a cell degradation pathway that eliminates damaged or unwanted proteins and organelles. Autophagy protects cells from chemotherapeutic agents by scavenging damaged mitochondria.

**Methods:**

Plasmid transfection and shRNA were used to regulate SHP-2 expression. Annexin V/PI staining were employed to analysis apoptosis. Flow cytometry was used to analyse intracellular calcium level and ROS. Immunofluorescence was used to detect mitochondria membrane potential, autophagy and Parkin translocation.

**Results:**

In cervical cancer, we found that SHP-2 suppressed apoptosis induced by Oxaliplatin and 5-FU. Further studies have found that SHP-2 protects against mitochondrial damage. This role of SHP-2 is associated with the activation of autophagy. In addition, SHP-2 degraded impaired mitochondria dependent on the ubiquitin ligase function of Parkin.

**Conclusions:**

These results suggest that SHP-2 inhibits the apoptosis induced by chemotherapeutic drugs through activating autophagy to degrade damaged mitochondria and ubiquitin ligase Parkin involved in SHP-2 induced autophagy.

## Background

Cervical cancer, the third most common malignant tumor, is mainly caused by human papillomavirus (HPV) infection [1]. Over 99% of cervical cancer cases are a result of HPV infection [2], and of those about 70% are a result of infection with HPV16/18 [3]. Tumor metastasis is an important cause of death in cancer patients. In patients with cervical cancer, lymph node metastasis reduced 5-year overall survival rates, compared with patients without lymph node metastasis [4]. Pelvic and abdominal aortic lymph node metastasis is common in advanced cervical cancer patients. Therefore, the aim of chemotherapy is to reduce tumor size, tumor growth and inhibit tumor metastasis in patients with cervical cancer.

In general, cytotoxic drugs are widely used in the treatment of tumors. 5-fluorouracil (5-FU), active cytotoxic drugs, inhibit the enzymatic activity of thymidylate synthase in DNA replication [5]. Oxaliplatin, another chemotherapeutic agent, inhibits tumor cell growth, causing cell stage G2 arrest and DNA covalent binding to form platinum-DNA adducts [6]. Chemotherapy is the most effective treatment for most cancer patients. However, drug resistance can cause tumor recurrence and reduce patient survival. Therefore, it is important for cancer patients to elucidate the mechanism of resistance to chemotherapy drugs.

In general, chemotherapy drugs can affect mitochondrial function. The characteristics of mitochondrial dysfunction is the loss of mitochondrial membrane potential, thereby causing the release of toxic reactive oxygen intermediates, leading to mitochondrial permeability transition pore opening, the release of cytochrome c and apoptosis [7–9]. Not surprisingly, cells have adaptive mechanisms, autophagy-that causes damaged mitochondria to be pinched off, eliminated, and protected by mitochondrial function. Autophagy is an important cytoplasmic process by recycling organelles as well as proteins to support normal cellular physiological functions during periods of stress. Classical autophagy is a selective accumulation of Parkin through the accumulation of PINK1 in the outer membrane of the mitochondrion, which ultimately promotes the degradation of dysfunctional mitochondria [10]. In summary, inhibition of autophagy contributes to the improvement of resistance to chemotherapeutic drugs.

SH2 domain-containing protein tyrosine phosphatase-2 (SHP-2) is one of the members of protein tyrosine kinase (PTK) family [11]. Some studies have shown that SHP-2 activation and mutation is closely related to the occurrence and development of malignant tumors. SHP-2 is overexpression in HPV infected cervical cancer patients. We have found that SHP-2 plays a critical role in mediating cervical cancer chemotherapeutic drugs resistance via degradation of dysfunctional mitochondria, and activation of the autophagy.

## Results

### *SHP*-*2 suppresses cervical cancer apoptosis induced by* Oxaliplatin and 5-FU

**Fig. 1:**
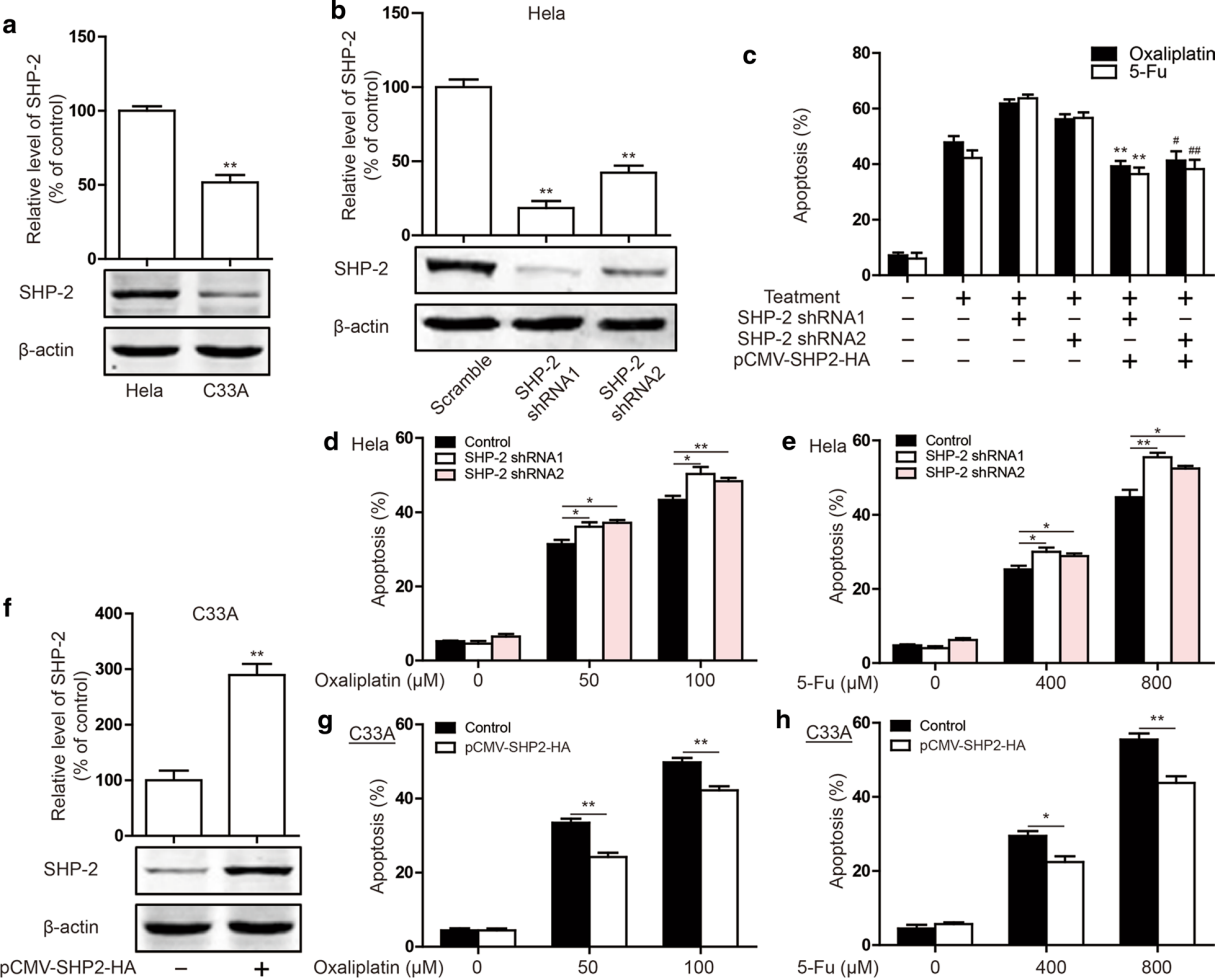
Effect of SHP-2 on apoptosis induced by Oxaliplatin and 5-FU in cervical cancer cells. **a** SHP-2 expression were detected by western blot in Hela cells and C33A cells. SHP-2 expression were detected by western blot in Hela cells, which were transfected SHP-2 shRNA1 or SHP-2 shRNA2 (**b**), and in C33A cells, which were transfected pCMV-SHP2-HA plasmid (**e**). Induction of apoptosis were measured by Annexin-V/PI double-staining assay in Hela cells, which were transfected SHP-2 shRNA1 or SHP-2 shRNA2 (**c**, **d**), and C33A cells, which were transfected pCMV-SHP2-HA plasmid (**f**, **g**) and treated different concentrations of Oxaliplatin (**c**, **f**) and 5-FU (**d**, **g**). Data are presented as mean ± SD. *P < 0.05, **P < 0.01

Studies have shown that SHP-2 expression is increased following HPV16/18 infection in cervical cancer. To confirm this conclusion, we selected the Hela (HPV18 +) and C33A (HPV-) cell line to examine the basic level of expression of SHP-2. As shown in Fig. 1a, the expression of SHP-2 was high in Hela cells, and low in C33A cells. Therefore, we selected the Hela and C33A cell line as the study object to investigate the influence of SHP-2 on the resistance to chemotherapy. Firstly, we examined the silencing efficiency of shRNA in Hela cell. As shown in Fig. 1b, SHP-2 expression was suppressed after transfection of SHP-2 shRNA1 and SHP-2 shRNA2. As expected, Oxaliplatin (Fig. 1c) and 5-FU (Fig. 1d) induced Hela cell apoptosis. Silencing SHP-2 promoted Hela cell apoptosis induced by these chemotherapeutic agents. Next, to investigate the effect of SHP-2 on chemotherapeutic induced apoptosis in C33A cells, the transfection of pCMV-SHP2-HA plasmid prompted SHP-2 to be expressed in C33A cells (Fig. 1e). Overexpression of SHP-2 reduced C33A cell apoptosis induced by Oxaliplatin (Fig. 1f) and 5-FU (Fig. 1g). The above results show that SHP-2 suppresses cervical cancer apoptosis induced by Oxaliplatin and 5-FU.

### Autophagy was involved in the inhibition of apoptosis by SHP-2

**Fig. 2:**
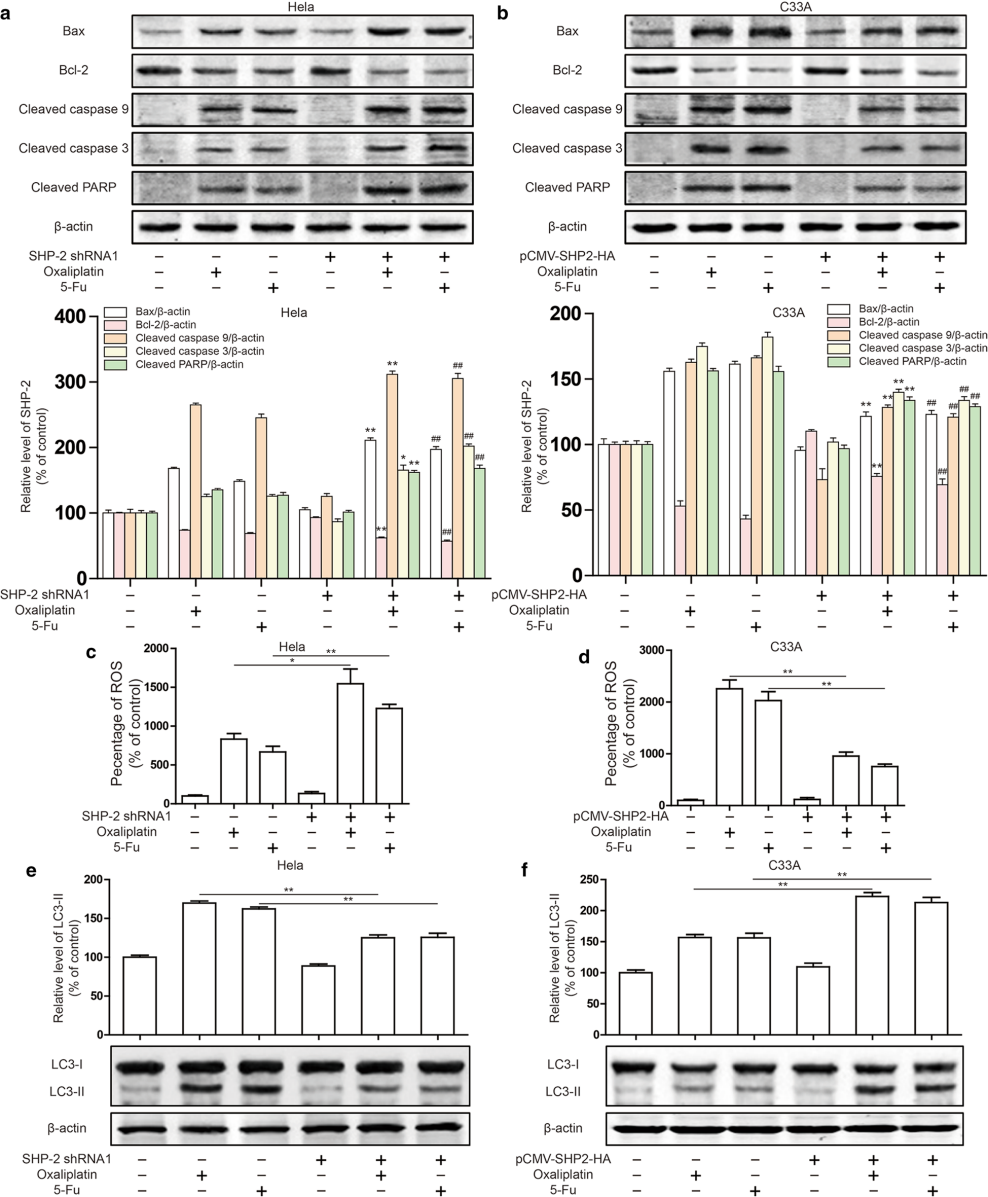
Effect of SHP-2 on the apoptosis induced by Oxaliplatin and 5-FU through autophagy. Cleaved caspases and cleaved PARP expression were detected by western blot in Hela cells (**a**), and C33A cells (**b**). Cells were transfected SHP-2 shRNA1 or pCMV-SHP2-HA plasmid and treated Oxaliplatin (100 μM) or 5-FU (800 μM). Data are presented as mean ± SD. *P < 0.05, **P < 0.01 compared with Oxaliplatin group. ^#^P < 0.05, ^##^P < 0.01 compared with 5-FU group. Intracellular ROS levels was measured by flow cytometry in Hela cells (**c**) and C33A cells (**d**). LC3 expression were detected by western blot in Hela cells (**e**) and C33A cells (**f**). Data are presented as mean ± SD. *P < 0.05, **P < 0.01

In order to further study the molecular mechanism of SHP-2 inhibiting apoptosis, we investigated the expression of apoptosis related proteins. As shown in Fig. 2a, Oxaliplatin and 5-FU induced bax, cleaved caspase 9, cleaved caspase 3 and cleaved PARP expression and reduced bcl-2 expression in Hela cell. Then, silencing SHP-2 can further promote bax, cleaved caspase 9, cleaved caspase 3 and cleaved PARP expression and repressed bcl-2 expression. In C33A cell, overexpression of SHP-2 reduced bax, cleaved caspase 9, cleaved caspase 3 and cleaved PARP expression and induced bcl-2 expression after Oxaliplatin and 5-FU treatment (Fig. 2b). Thus, SHP-2 improved the mitochondrial apoptosis induced by Oxaliplatin and 5-FU. Furthermore, the changes of cell ROS level were further investigated, and it was found that the silencing SHP-2 could raise the ROS level in Hela cells, while overexpressing SHP-2 could reduce the ROS level in C33A cells (Fig. 2c, d). The changes of ROS are inseparable from mitochondrial involvement, while autophagy effectively protects the mitochondrial function. Then the changes in autophagy levels were studied. Silencing SHP-2 could down-regulated LC3-II expression in Hela cells, while overexpressing SHP-2 could up-regulated LC3-II expression in C33A cells (Fig. 2e, f). These results suggest that SHP-2 inhibits apoptosis induced by chemotherapeutic drugs by autophagy.

### SHP-2 protects mitochondria from damage

**Fig. 3:**
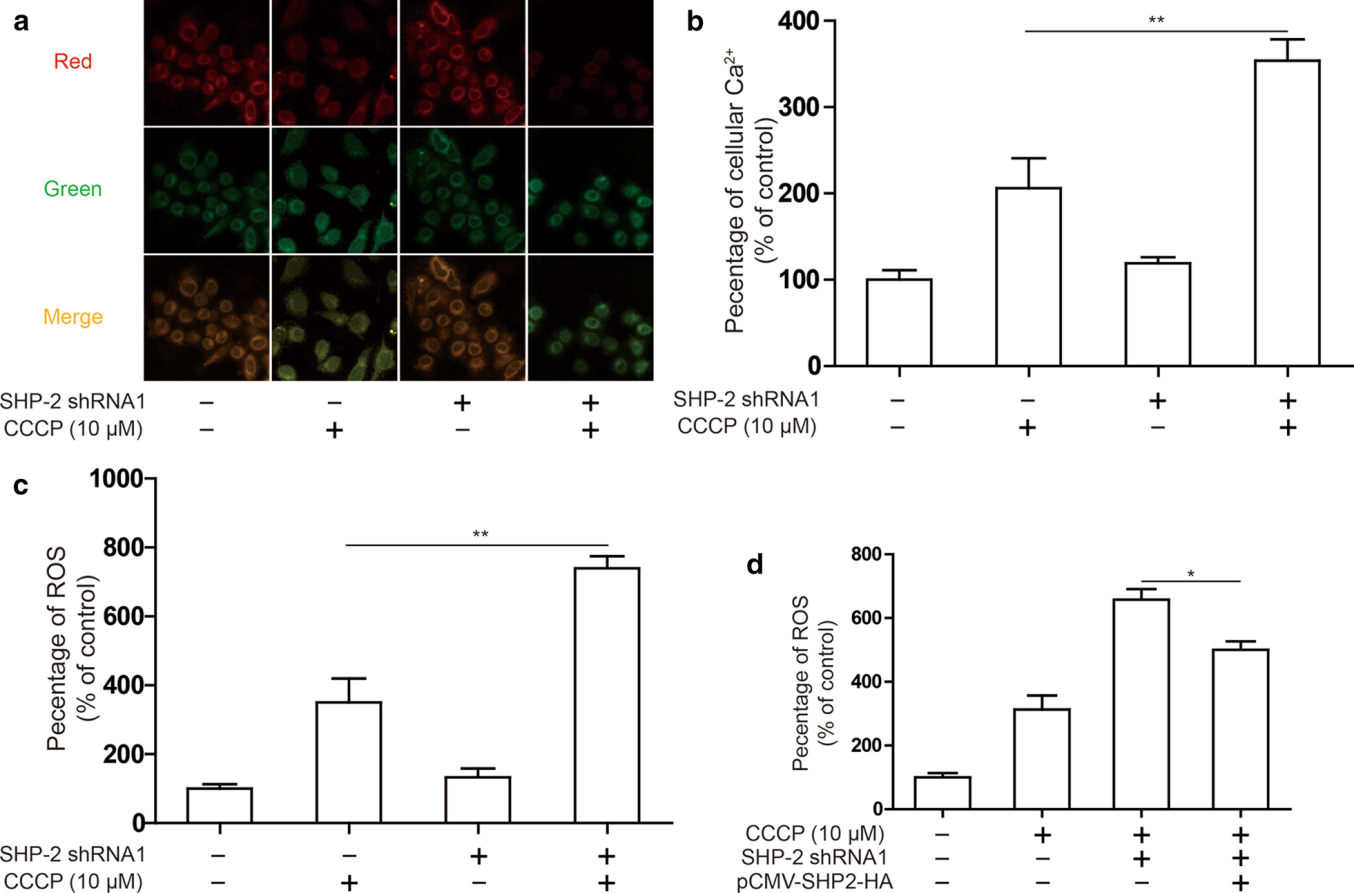
Effect of SHP-2 on mitochondria damage in cervical cancer cells. Hela cells were transfected SHP-2 shRNA1 and treated CCCP (10 μM). **a** The change of ΔΨm was detected by confocal laser scanning microscope using JC-1 staining. **b** The Ca^2+^ level in cytoplasm was detected by flow cytometry. **c**, **d** Intracellular ROS levels was measured by flow cytometry. Data are presented as mean ± SD. **P < 0.01

To investigate the mechanism by which SHP-2 inhibits apoptosis induced by Oxaliplatin and 5-FU, we investigated the effects of SHP-2 on mitochondrial function. Usually, the action of chemotherapeutic drugs can lead to the loss of mitochondrial membrane potential, resulting in the release of toxic reactive oxygen intermediates, leading to the release of cytochrome c and apoptosis. To test whether SHP-2 affects mitochondrial damage, we measured mitochondrial membrane potential in Hela cell. CCCP treatment induced loss of mitochondrial membrane potential, and this was exacerbated after diminishing SHP-2 expression (Fig. 3a). Similar findings that CCCP treatment increased cellular Ca^2+^ level and this was aggravated after diminishing SHP-2 expression (Fig. 3b). The release of toxic reactive oxygen species was further examined, and the results showed that CCCP could up-regulate the ROS level. Silencing SHP-2 increased the ROS level in Hela cell induced by CCCP (Fig. 3c, d). These results suggested that SHP-2 protects mitochondria from damage in Hela cell.

### SHP-2 promotes cervical cancer autophagy activation

**Fig. 4:**
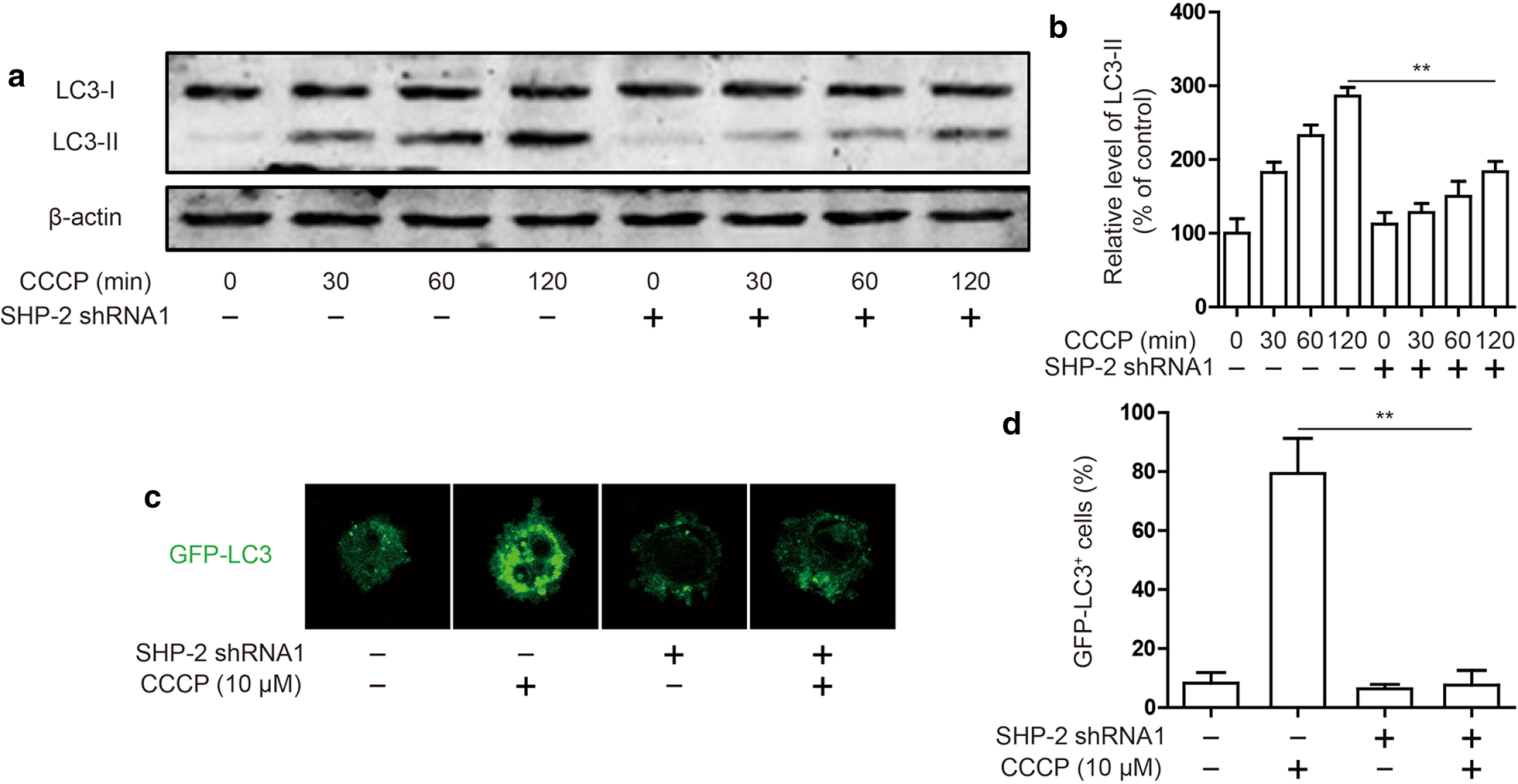
Effect of SHP-2 on autophagy activation induced by CCCP in Hela cells. **a**, **b** LC3 expression were detected by western blot. Hela cells were transfected SHP-2 shRNA1 and treated CCCP (10 μM) for different time. Data are presented as mean ± SD. **P < 0.01. Induction of GFP + dots in Hela cells expressing GFP-LC3. Cells were transfected SHP-2 shRNA1 and treated CCCP (10 μM). Images of individual cells **c** and quantified of GFP-LC3 + dots (**d**). Data are presented as mean ± SD. **P < 0.01

To further study the mechanism of SHP-2 prevented mitochondrial damage, we hypothesized that autophagy is involved in this process. The changes in LC3 expression under CCCP stimulation were examined by western blots. As expected, the expression of LC3-II was up-regulated in different times of CCCP stimulation. Silencing SHP-2 inhibited LC3-II expression induced by these CCCP in Hela cell (Fig. 4a, b). Additionally, diminishing SHP-2 expression decreased GFP-LC3 puncta accumulation after treated with CCCP (Fig. 4c, d). The data indicated that silencing SHP-2 suppressed autophagy activation induced by CCCP in Hela cell.

**Fig. 5:**
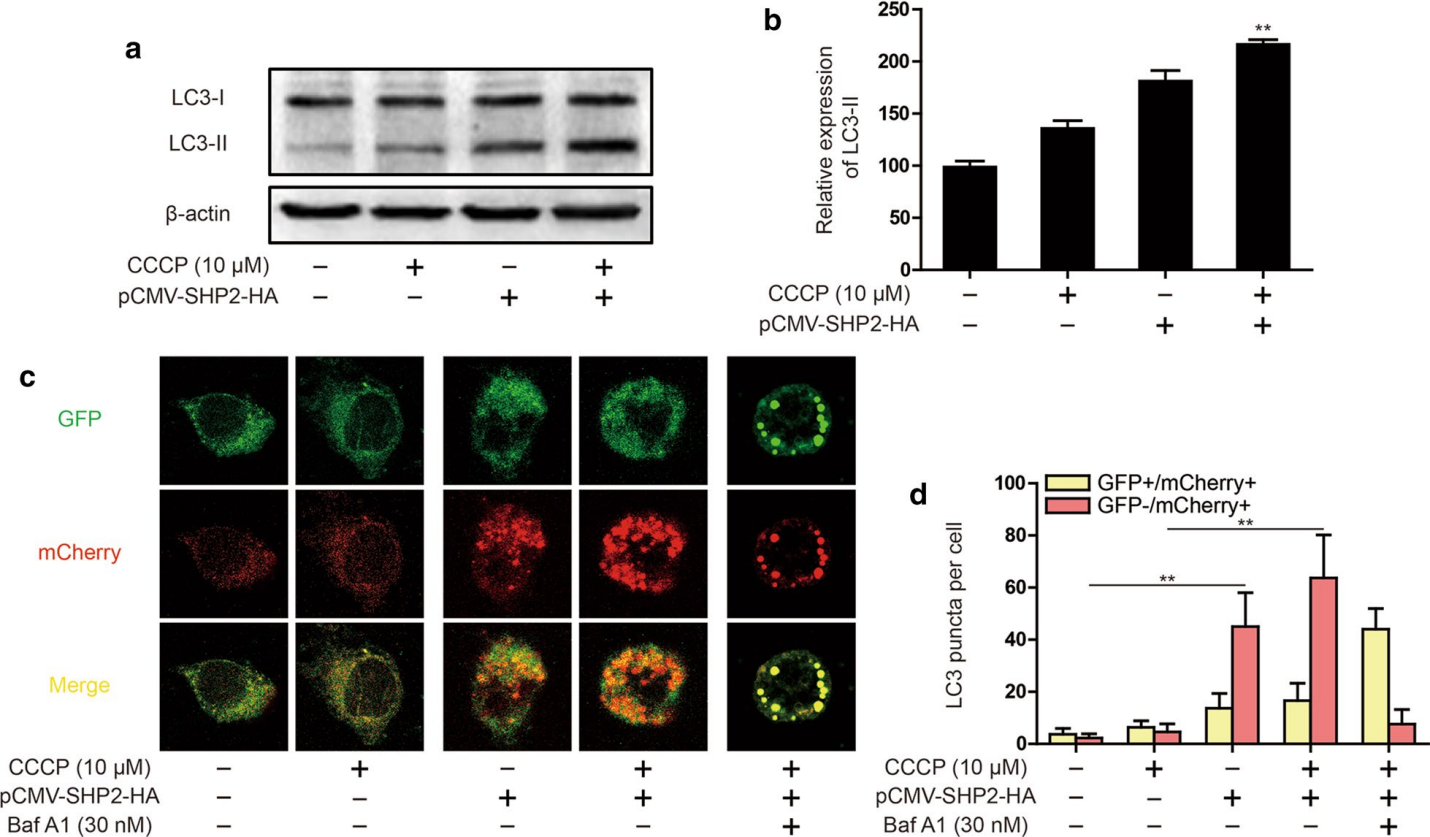
Effect of SHP-2 on autophagy activation induced by CCCP in C33A cells. **a**, **b** LC3 expression were detected by western blot. C33A cells were transfected pCMV-SHP2-HA plasmid and treated CCCP (10 μM). Data are presented as mean ± SD. **P < 0.01 compared with CCCP treatment group. **c** Expressing mCherry-GFP-LC3 C33A cells were observed under a confocal microscopy after pCMV-SHP2-HA plasmid transfection or CCCP (10 μM) and Baf A1 (30 nM) treatment. Representative images are shown. **d** The average numbers of yellow or red puncta were obtained from three countings. Data are presented as mean ± SD. **P < 0.01 compared with CCCP treatment group

Next, we further investigated the effect of SHP-2 on autophagy activation in C33A (HPV-) cell. Overexpression of SHP-2 not only up-regulated the basal level of LC3-II, but also increased the expression of LC3-II induced by CCCP (Fig. 5a, b). To further assess the status of autophagic flux, we used the mCherry-GFP-LC3 construct. Overexpression of SHP-2 increased the numbers of GFP-mCherry + (red) puncta, indicating an increase in autolysosomes. In contrast, large-sized GFP + mCherry + (yellow) puncta and few GFP-mCherry + (red) puncta were observed in bafilomycin A1 treatment group (Fig. 5c, d). This demonstrated overexpression of SHP-2 promoted autophagy activation in C33A (HPV-) cells.

### SHP-2 involves in the clearance of damaged mitochondria

**Fig. 6:**
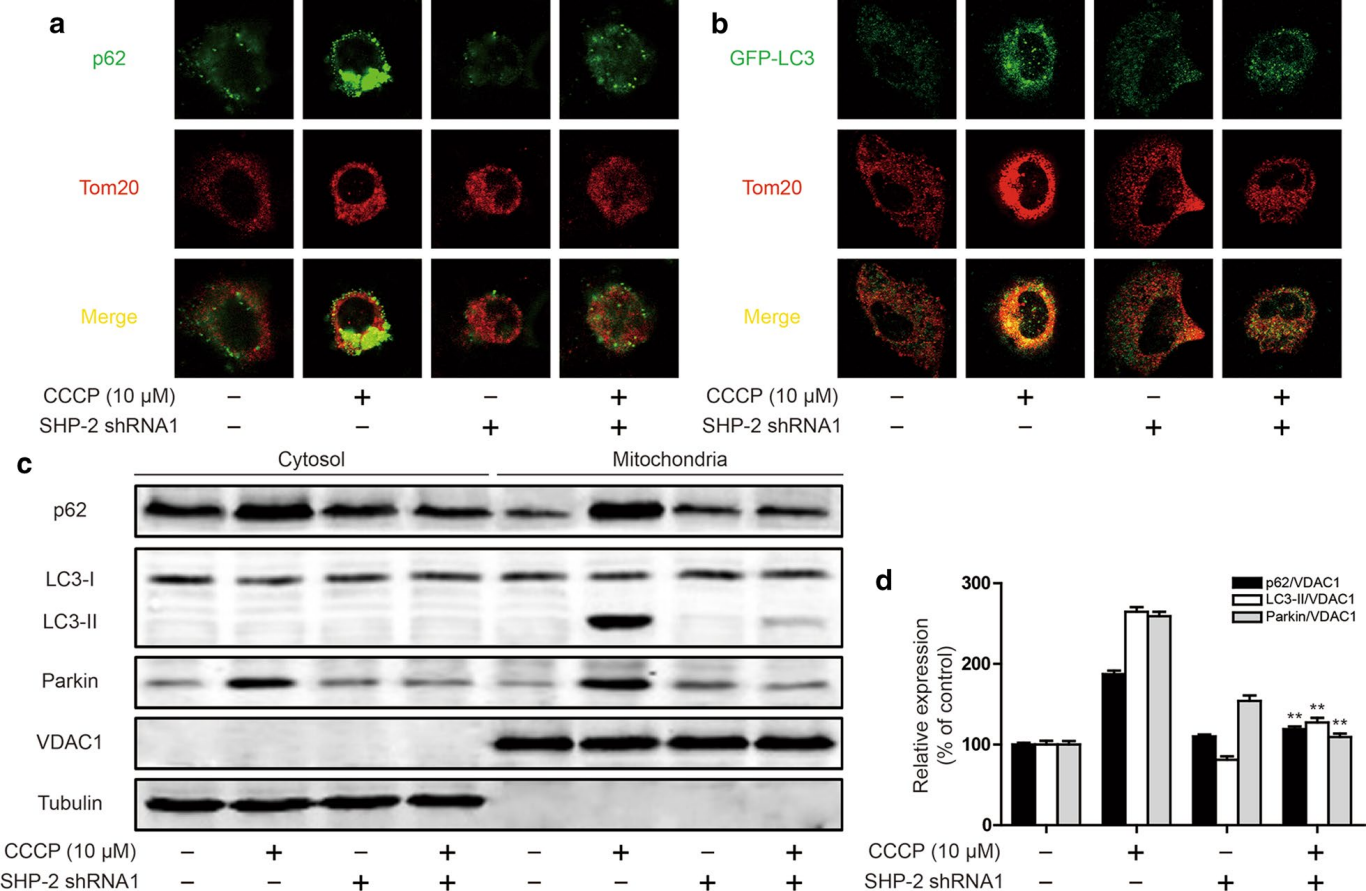
Effect of SHP-2 on clearance of damaged mitochondria. **a** Confocal microscopy of Hela cells transfected SHP-2 shRNA1 or treated CCCP (10 μM), immunostained for p62 (green) and Tom20 (red). **b** Confocal microscopy of GFP-LC3 + Hela cells transfected SHP-2 shRNA1 or treated CCCP (10 μM), immunostained for endogenous Tom20. **c, d** Subcellular distribution of p62, LC3-II, and Parkin a transfected SHP-2 shRNA1 or treated CCCP (10 μM). Data are presented as mean ± SD. **P < 0.01 compared with CCCP treatment group. Data are representative of at least three experiments

To further investigate the mechanism of SHP-2 in protecting mitochondrial damage, we examined the effect of SHP-2 on clearance of damaged mitochondria. We therefore examined whether SHP-2 affect the subcellular distribution of p62 and/or induce its mitochondrial translocation. In non-stimulated Hela cell, p62 displayed diffuse cytoplasmic distribution, but CCCP induced p62-containing aggregates that were colocalized with mitochondria. Silencing SHP-2 suppressed p62-containing aggregates and -translocating to mitochondria (Fig. 6a). Similar results showed that CCCP also induced GFP-LC3 colocalized with mitochondria, however, silencing SHP-2 suppressed GFP-LC3 translocation to mitochondria (Fig. 6b). Cell fractionation confirmed these results. In non-stimulated Hela cell, very little p62 and LC3-II co-sedimented with mitochondria, but after incubation with CCCP, much more p62 and LC3-II was present in the mitochondrial fraction, however, p62 and LC3-II was vanishing in the mitochondrial fraction after silencing SHP-2 (Fig. 6c, d). These results suggest that SHP-2 involved in translocation of p62 and LC3 to mitochondria.

### SHP-2 regulates the clearance of damaged mitochondria dependent on the ubiquitin ligase function of Parkin

**Fig. 7:**
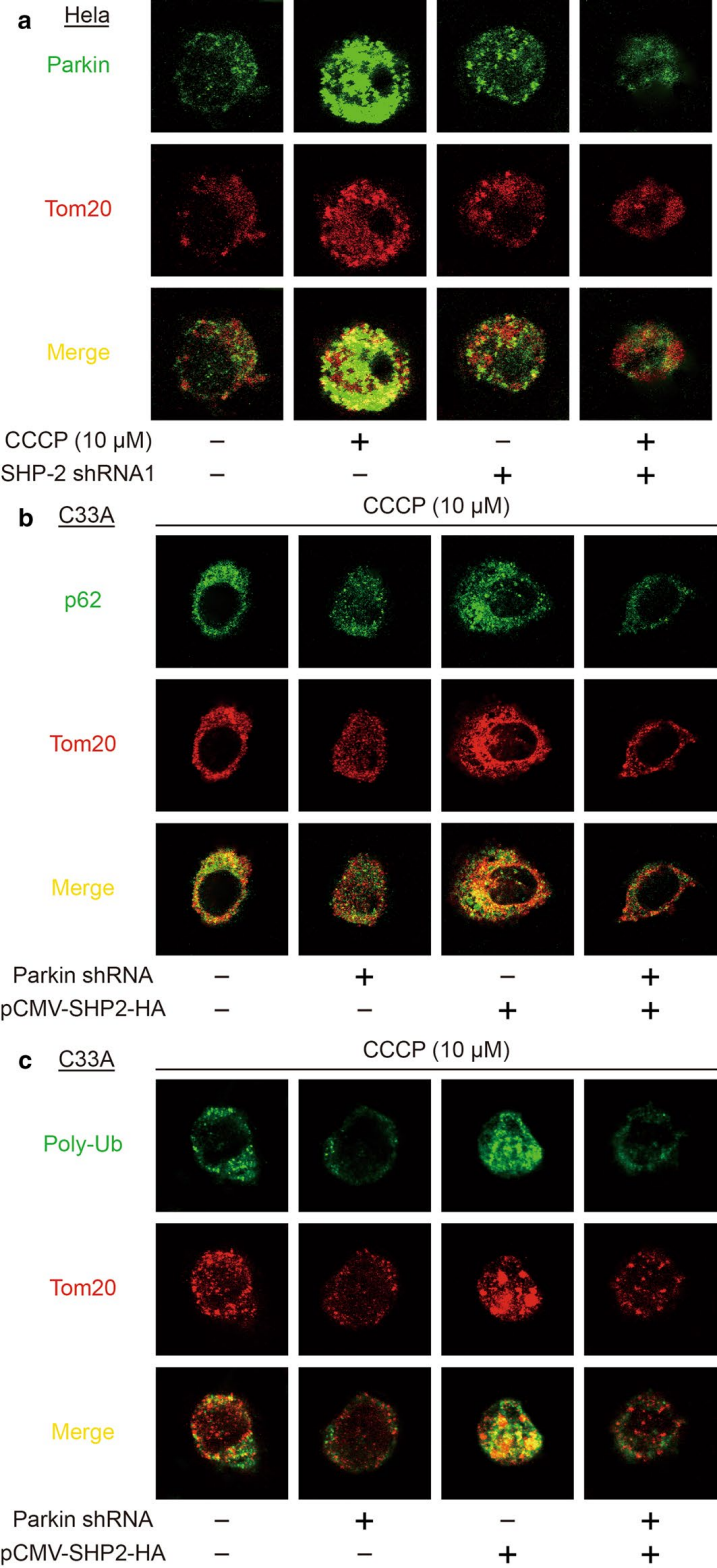
The clearance of damaged mitochondria is Parkin-dependent. **a** Confocal microscopy of Hela cells transfected SHP-2 shRNA1 or treated CCCP (10 μM), immunostained for Parkin (green) and Tom20 (red). **b** Confocal microscopy of C33A cells transfected Parkin shRNA or pCMV-SHP2-HA plasmid, immunostained for p62 (green) and Tom20 (red). **c** Confocal microscopy of C33A cells transfected Parkin shRNA or pCMV-SHP2-HA plasmid, immunostained for Poly-Ub (green) and Tom20 (red). Data are representative of at least three experiments

The canonical pathway for *the clearance of damaged mitochondria* by autophagy involves Parkin recruitment to the mitochondrion. In Fig. 6c and d, Parkin was also translocated into mitochondria after incubation with CCCP. Silencing SHP-2 inhibited Parkin presented in the mitochondrial fraction. Further studies showed that CCCP induced Parkin colocalized with mitochondria. Silencing SHP-2 suppressed Parkin colocalized with mitochondria in Hela cell (Fig. 7a). Moreover, silencing Parkin reduced p62 and mitochondria co-localization induced by CCCP and SHP-2 overexpression in C33A cell (Fig. 7b). Induction of mitochondrial poly-ubiquitination is likely to depend on the E3 ligase Parkin [6]. Indeed, silencing Parkin reduced mitochondrial poly-ubiquitination induced by CCCP and SHP-2 overexpression in C33A cell (Fig. 7c). Thus, SHP-2 regulates the clearance of damaged mitochondria dependent on the ubiquitin ligase function of Parkin.

## Discussion

Capecitabine (5-FU) combined with platinum based chemotherapy has been widely used in the treatment of various types of cancer [12]. Although the initial response to surgery and chemotherapy in patients with cervical cancer is often effective, resistant carcinomas usually occur with patients who die of disease recurrence [13, 14]. Therefore, understanding the mechanisms of chemoresistance in cervical cancer is critical for optimizing current treatment strategies.

The mechanism of chemotherapeutic drugs cannot be separated from the induction of apoptosis. Dysfunction of mitochondrial function is the beginning of apoptosis induction. Mitochondrial dysfunction is manifested by a decrease in mitochondrial membrane potential, resulting in increased permeability of the mitochondrial membrane, resulting in the release of ROS, cytochrome c and ultimately apoptosis. Therefore, if the mitochondrial function is damaged, if it is not removed in time, it will damage the tissues of the body. In the cell, autophagy activation will remove damaged mitochondria.

SHP-2, as a subset of the protein tyrosine phosphatase family [15, 16], is an important role in cell proliferation, apoptosis, differentiation and migration, and is also the downstream signal molecules of various cytokines, extracellular matrix and antigen gene. The literature showed that SHP-2 is overexpression in HPV infected cervical cancer patients. Thus, the degree of malignancy of cervical cancer is related to HPV infection. Therefore, studying the role of SHP-2 in cervical cancer is of great significance. Through our study, we found that SHP-2 can induce chemoresistance of cervical cancer. Further studies have shown that SHP-2 protects against mitochondrial damage in cervical cancer cells. Finally, the mechanism of SHP-2 was investigated, and it was found that autophagy could protect the normal physiological functions of cervical cancer cells by activating autophagy to remove damaged mitochondria. Damaged mitochondria are recognized by the E3 ubiquitin ligase Parkin, which decorates their outer membrane proteins with poly-ubiquitination chains [17]. p62 bind to poly-ubiquitination chains through its UBA domain to autophagic clearance [18]. Therefore, we investigated whether Parkin is involved in SHP-2 induced autophagy. After research found that SHP-2 promotes the destruction of damaged mitochondria by activating the Parkin signaling pathway.

In summary, SHP-2 protects cells from the destruction of chemotherapeutic drugs by activating autophagy to remove damaged mitochondria. Its molecular mechanism is to degrade mitochondria via the ubiquitin ligase of Parkin. These studies preliminarily illustrate the influence of SHP-2 on autophagy. But there is a need for comprehensive validation in vivo.

## Conclusion

In present study, we discovered that SHP-2 suppressed Oxaliplatin and 5-FU induced apoptosis through protecting against mitochondrial damage. Further autophagy was involved in SHP-2 function. SHP-2 degraded impaired mitochondria dependent on the ubiquitin ligase function of Parkin. These results indicate that SHP-2 inhibits the apoptosis induced by chemotherapeutic drugs through activating autophagy to degrade damaged mitochondria and ubiquitin ligase Parkin involved in SHP-2 induced autophagy.

## Materials and methods

### Reagents

Oxaliplatin, 5-FU and CCCP were purchased from Sigma-Aldrich (St. Louis, USA). Dye DAPI was purchased from Invitrogen (Carlsbad, USA). Paraformaldehyde (PFA) was purchased from Yonghua Chemical Technology (Jiangsu) Co. Ltd. (Changshu, China). Triton X-100 was purchased from Shanghai Chao Rui Biotech. Co. Ltd. (Shanghai, China). BSA was purchased from Roche Diagnosis (Shanghai) Ltd. (Shanghai, China).

### Antibodies

Primary antibodies against caspase 3, caspase 9 and β-actin were obtained from Santa Cruz Biotechnology (CA, USA); LC3, Bax and Bcl-2 was from Bioworld (OH, USA) and antibodies against PARP, SHP-2, VDAC1, Tubulin, Tom20, Parkin and Poly-Ub were purchased from Cell Signaling Technology (Danvers, MA); Antibodies to SQSTM1/p62 were obtained from Abcam (Cambridge, UK). IRDye™ 800 conjugated secondary antibodies were obtained from Rockland Inc. (Philadelphia, USA). Alexa Fluor 488 donkey anti-rabbit IgG, Alexa Fluor 594 donkey anti-mouse IgG were obtained from Invitrogen (CA, USA).

### Cell culture

Human cervical cancer cell lines Hela (HPV18-positive) and C33A (HPV-negative) were purchased from the American Type Culture Collection (ATCC, USA). All cells were cultured in Dulbecco’s Modified Eagle Medium (Gibco, USA) containing 10% fetal bovine serum at 37 °C in 5% CO_2_.

### Annexin V/PI staining

Cells were harvested, washed and resuspended in PBS, then stained with the Annexin V/PI Cell Apoptosis Detection Kit (KeyGen Biotech, Nanjing, China) according to the manufacturer’s instructions. Data acquisition and analysis were performed with a Becton–Dickinson FACS Calibur flow cytometer using Cell-Quest software (BD Biosciences, Franklin Lakes, NJ). The cells in early stages of apoptosis were Annexin V positive and PI negative, whereas the cells in the late stages of apoptosis were both Annexin V and PI positive.

### Mitochondrial transmembrane potential (*ΔΨ*_*m*_) assessment

The electrical potential difference across inner *ΔΨ*_*m*_ was monitored using the *ΔΨ*_*m*_-specific fluorescent probe JC-1 (Beyotime Institute of Biotechnology, China). The *ΔΨ*_*m*_-specific fluorescent probe JC-1 (Beyotime Institute of Biotechnology, China) exists as a monomer with an emission at 530 nm (green fluorescence) at low membrane potential but forms J-aggregates with an emission at 590 nm (red fluorescence) at higher potentials. Cells were harvested and incubated with JC-1 for 30 min at 37 °C in the dark, then resuspended in washing buffer and photographed with a confocal laser scanning microscope (Fluoview FV1000, Olympus, Tokyo, Japan).

### Intracellular calcium level assessment

Cells were loaded with 10 mM Fluo-3 AM (Beyotime Institute of Biotechnology, China) which combined with Ca^2+^ and produced strong fluorescence. After 30 min incubation at 37 °C in the dark, the cells were washed with PBS twice and the fluorescence intensity was measured by FACSCalibur flow cytometry (Becton–Dickinson) at Ex./Em. − 488/525 nm.

### Measurement of reactive oxygen species formation (ROS)

The level of ROS was detected using fluorescent dye 2,7-dichlorofluorescein-diacetate (DCFHDA, Beyotime Institute of Biotechnology, China). Levels of ROS was detected using fluorescent dye 2,7-dichlorofluorescein-diacetate (DCFHDA, Beyotime Institute of Biotechnology, China). Cells were collected and incubated with DCFH-DA for 30 min at 37 °C in the dark. The fluorescence intensity was measured using flow cytometry (FACSCalibur, Becton–Dickinson).

### Mitochondrial fractionation

Mitochondrial fractionation kit (KeyGen Biotech, China) was used to get mitochondrial according to the following protocol. Cells were incubated 3.5 × 10^7^ cells/1 mL ice-cold mitochondrial lyses Buffer, then suspended and ground the cells with tight pestle on ice. The homogenate was subjected to centrifuging at 800 g for 5 min at 4 °C to remove nuclei and unbroken cells, and then added 0.5 mL supernatant above the 0.5 mL Medium Buffer in the new 1.5 mL tube gently. After centrifugation at 15,000*g* for 10 min at 4 °C, the supernatant was carefully removed and collected as the cytosolic fraction and the remaining mitochondrial pellet was resuspended in the mitochondrial extraction buffer.

### Cell transfection

GFP-LC3, mCherry-GFP-LC3 plasmid and pCMV-SHP2-HA plasmid (Addgene, MA, USA) and the shRNA targeting human SHP-2 or human Parkin, or control shRNA with scrambled sequence (Santa Cruz, CA, USA) were transfected using Lipofectamine 2000™ reagent (Invitrogen, CA, USA), according to the manufacturer’s instructions 50.

### Western blot analysis

Western blot analysis was prepared as described previously (25). Protein samples were separated by 10% SDS-PAGE and transferred to onto nitrocellulose membranes. The membranes were blocked with 1% BSA at 37 °C for 1 h and incubated with indicated antibodies overnight at 4 °C, followed by IRDye800 conjugated secondary antibody for 1 h at 37 °C. Immunoreactive protein was detected with an Odyssey Scanning System (LI-COR Inc., Lincoln, Nebraska).

### Immunofluorescence

Cells were washed with PBS, fixed with 4% paraformaldehyde and washed again with PBS. Nonspecific receptors on cells were blocked for 1 h with 3% BSA. Rabbit anti-p62 (Abcam, Cambridge, UK), Rabbit anti-Parkin (CST, MA, USA), Rabbit anti-Poly-Ub (CST, MA, USA), mouse anti-Tom20 (CST, MA, USA) were used for immunostaining. Alexa Fluor 488 donkey anti-rabbit IgG, Alexa Fluor 594 donkey anti-mouse IgG were used as secondary antibodies (Invitrogen, CA, USA). Samples were observed and captured with a confocal laser scanning microscope (Olympus Corp., Tokyo, Japan).

### Statistical analysis

The data shown in the study were obtained in at least three independent experiments and all results represent the mean ± S.E.M. Differences between the groups were assessed by one-way ANOVA test. Details of each statistical analysis used are provided in the figure legends. Differences with P values < 0.05 were considered statistically significant.

## Abbreviations

SHP-2: SH-2-containing phosphatase
ROS: reactive oxygen species
HPV: human papillomavirus
PINK1: PTEN induced putative kinase 1
PTK: protein tyrosine kinase
CCCP: carbonyl cyanide *m*-chlorophenylhydrazone
5-FU: 5-fluorouracil
LC3: microtubules associated protein 1 light chain3-beta
